# Supplementary material for: Cisplatin toxicity is counteracted by the activation of the p38/ATF-7 signaling pathway in post-mitotic C. elegans
Source: Nat Commun. 2023 May 20;14:2886. doi: 10.1038/s41467-023-38568-5 (PMC10199892; doi:10.1038/s41467-023-38568-5)
Supplement: Supplementary file 1 — Supplementary Information [file 41467_2023_38568_MOESM1_ESM.pdf]

Cisplatin toxicity is counteracted by the activation of p38/ATF-7 signaling pathway in post-mitotic *C. elegans*

Dorota Raj[1], Bashar Kraish[1], Jari Martikainen[2], Agnieszka Podraza-Farhanieh[1,4], Gautam Kao[1,*] and Peter Naredi,[1,3,*]

[1]Department of Surgery, Institute of Clinical Sciences, Sahlgrenska Academy, University of Gothenburg, SE413 45 Gothenburg, Sweden

[2]Bioinformatics and Data Centre, Sahlgrenska Academy, University of Gothenburg, Gothenburg, SE413 45 Gothenburg, Sweden.

[3]Department of Surgery, Sahlgrenska University Hospital, SE413 45 Gothenburg, Sweden

[4]Current address: Lundberg Laboratory for Diabetes Research, Department of Molecular and Clinical Medicine, Sahlgrenska Academy, University of Gothenburg, SE413 45 Gothenburg, Sweden.

*Corresponding authors: peter.naredi@gu.se; gautam.kao@gu.se

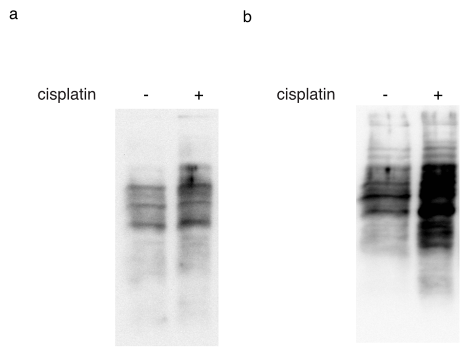

Supplementary Figure 1. The influence of cisplatin exposure on protein oxidation was evaluated using the OxyBlot kit. Proteins were recovered from lysates of worms exposed to cisplatin plates for 3h at concentrations of (a) 300µg/mL and (b) 600µg/mL. Proteins in the lysates were labelled with DNP solution (Oxyblot) to reveal the presence of protein carbonyl modifications. The experiment was performed in one replicate for both conditions.

Blots used for quantification of phospho-p38 levels in **Figure 1b**

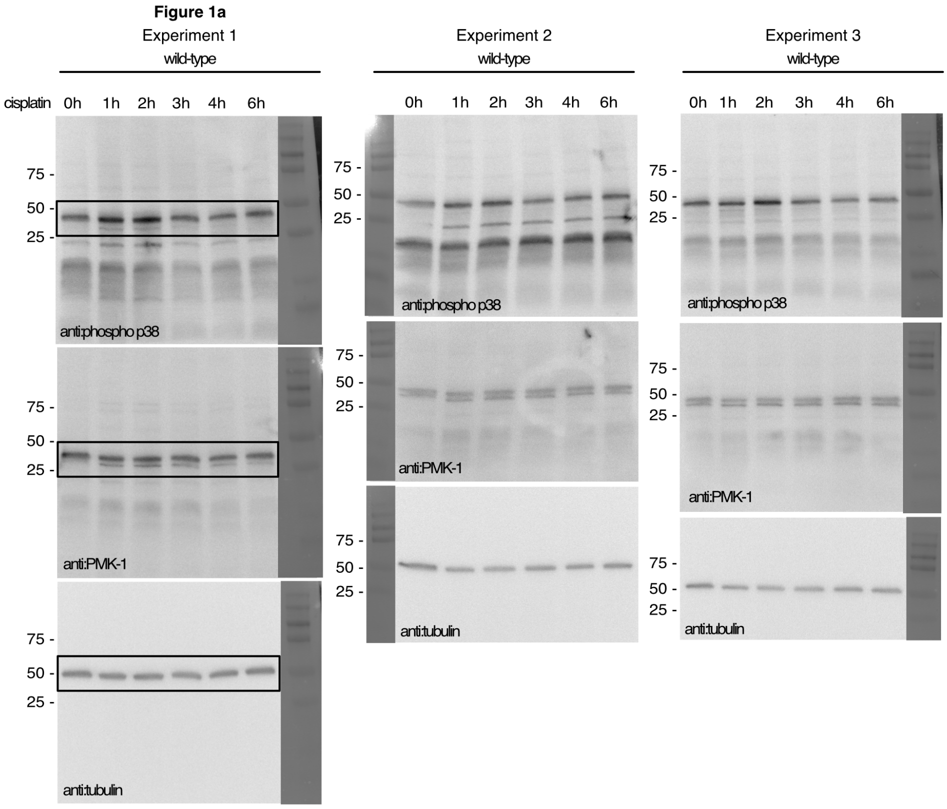

Blots used for quantification of phospho-p38 levels in **Figure 1d**

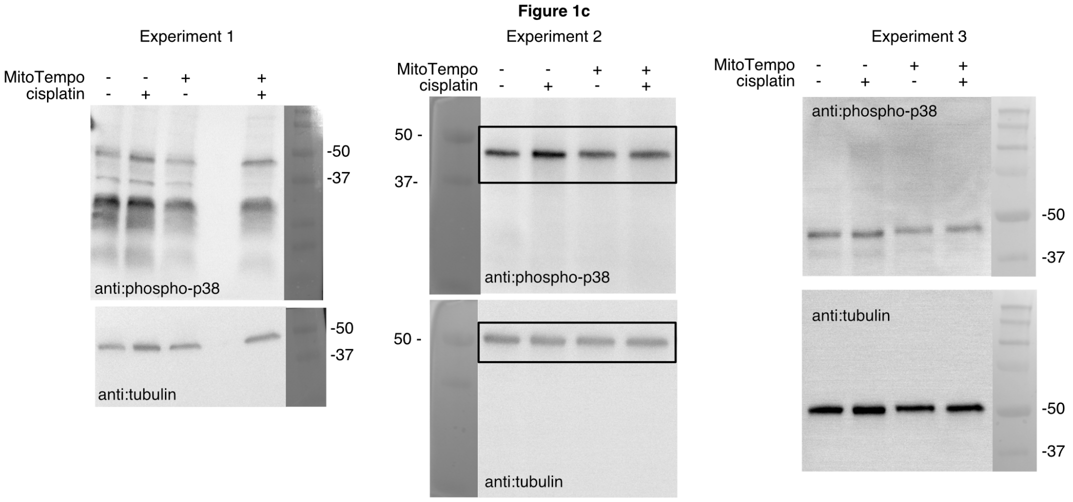

Full blot images

Supplementary Figure 2. Full blot images.

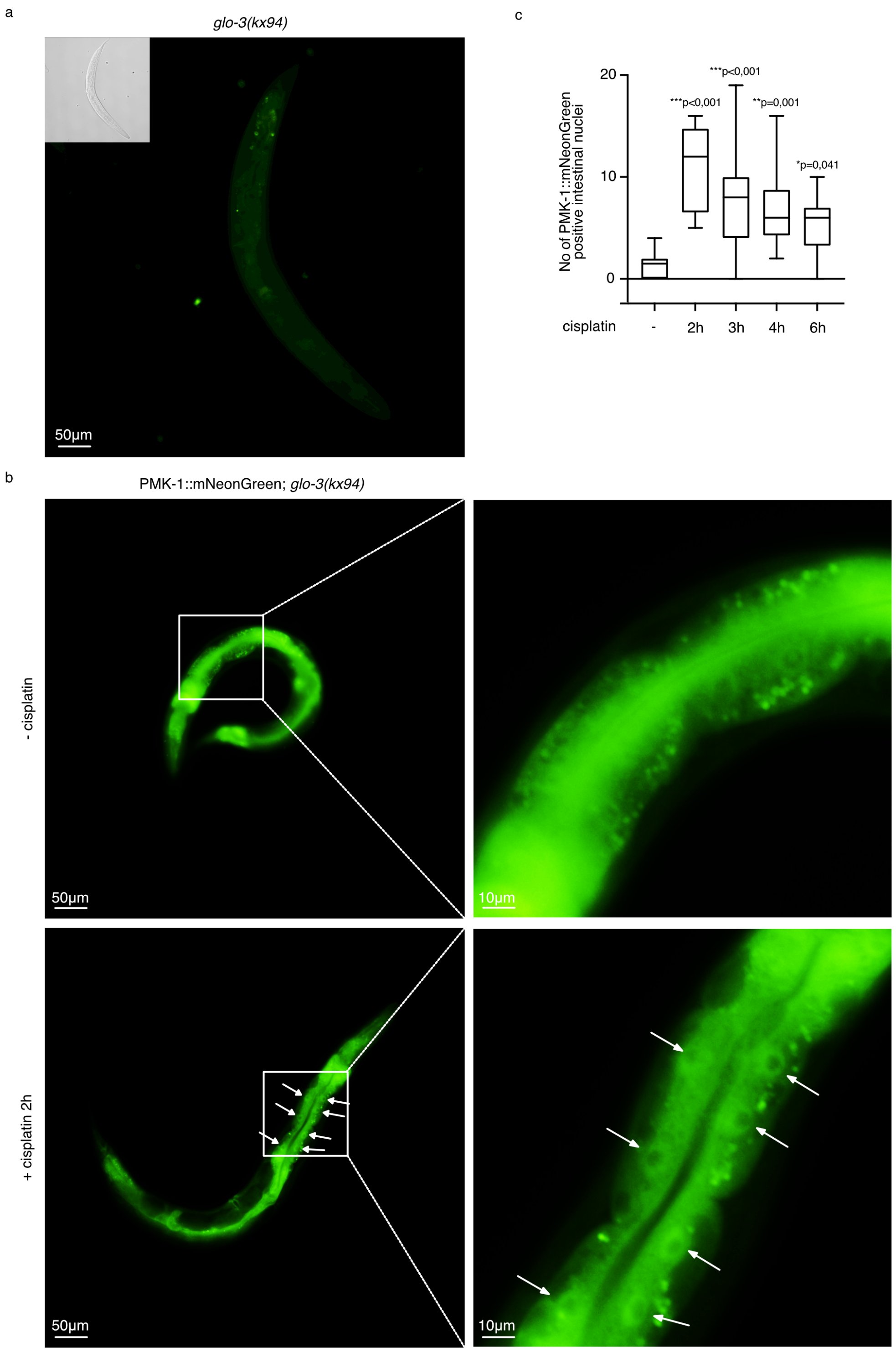

Supplementary Figure 3. (a) Representative fluorescent pictures of *glo3-(kx94)*, which partially lacks autofluorescent granules in intestinal cells. (b) Representative fluorescent pictures of localization of PMK-1::mNeonGreen (*re170)* in the *glo-3(kx94)* background in 1-day-old animals untreated or treated with cisplatin solution (450 µg/mL for 2 h). Increased numbers of intestinal cell nuclei containing PMK-1::mNeonGreen after cisplatin treatment were observed. The white square in the left panels marks the area magnified in the right panels. In the right panels, white arrows indicate PMK-1::mNeonGreen accumulation in intestinal nuclei. (c) The estimation of a number of PMK-1::mNeonGreen positive nuclei in intestinal cells in untreated (n=12) and cisplatin-treated (450µg/mL for 2 h ((n=12), 3 h

(n=15), 4 h (n=12), and 6 h (n=12)) 1-day old animals. Box plots indicate median (middle line), 25th, 75th percentile (box) and Min to Max (whiskers). Statistical significance was determined by one-way ANOVA followed by Bonferroni post hoc correction. The lack of green fluorescence in *glo-3* mutants in (a) shows that the green fluorescence detected in the *pmk-1::mNeonGreen;glo-3(kx94)* strain is from the transgene. Source data are provided as a Source Data file.

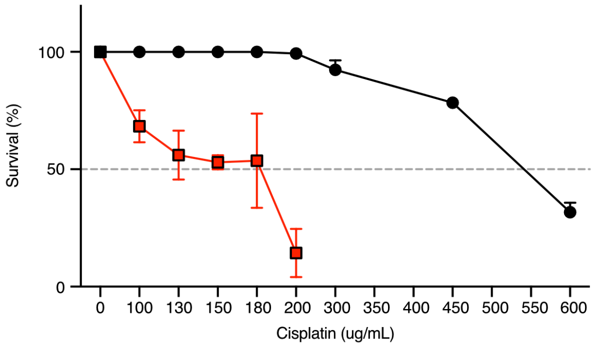

Supplementary Figure 4. LD_{50} determination for cisplatin-induced death in *sek-1(km4)* mutants (red squares) and wild-type animals (black circles) after exposure for 24 h at varying concentrations. The experiment was performed in triplicate. Bars represent mean ± SD. Source data are provided as a Source Data file.

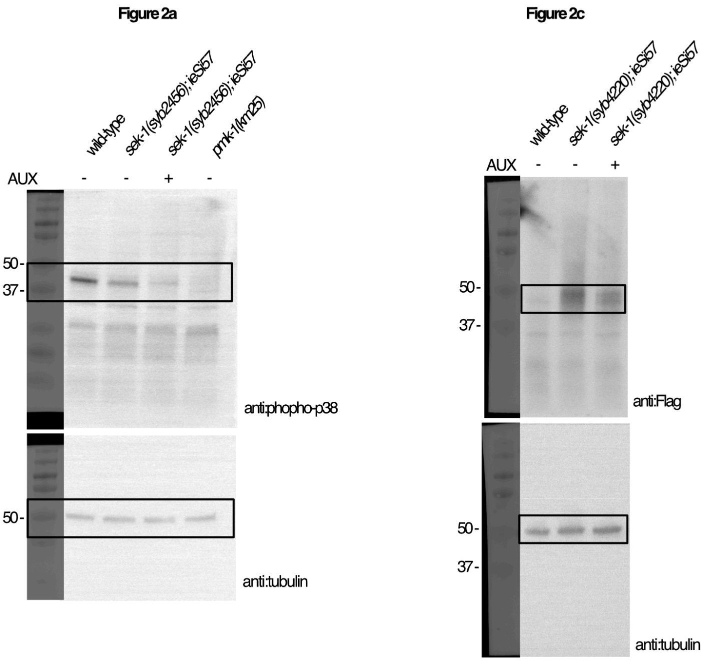

Supplementary Figure 5. Full blot images.

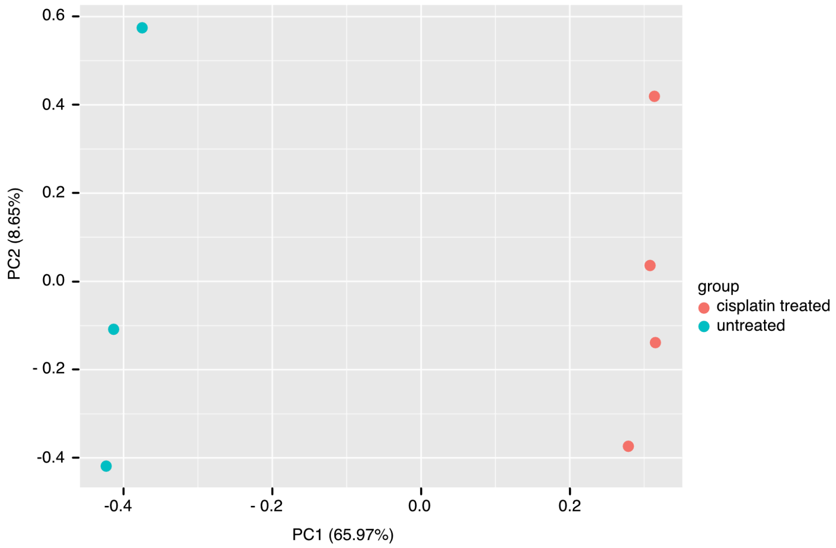

Supplementary Figure 6. Principal component analysis (PCA) of the proteomics datasets from 1-day-old adult wild-type animals untreated or cisplatin-treated (300 µg/mL) for 6 h.

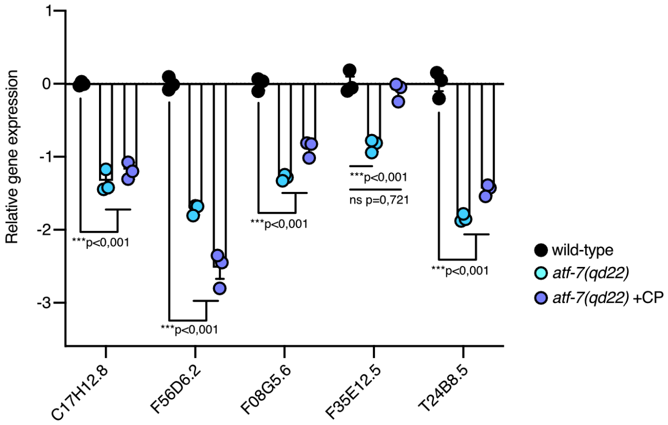

Supplementary Figure 7. Relative gene expression of PMK-1-dependent immune genes in 1-day-old adult animals of the indicated genotypes without or with (+CP) cisplatin treatment. Worms were exposed for 6 h to cisplatin on plates containing 300 µg/mL cisplatin. Statistical significance was determined by one-way ANOVA followed by Bonferroni post-hoc correction. The experiment was performed in triplicate. F44B9.5 was used as a normalizing control. Bars represent mean ± SEM. Source data are provided as a Source Data file.

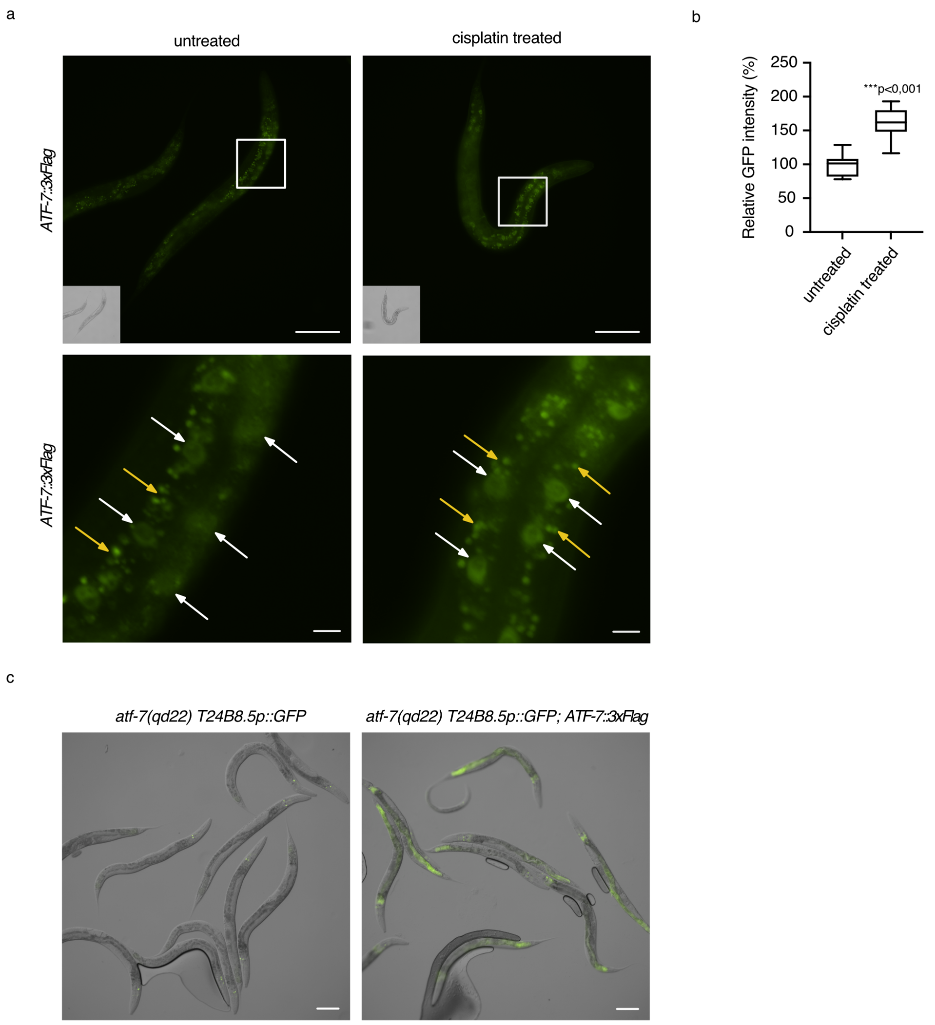

Supplementary Figure 8. (a) Representative fluorescent pictures of ATF-7::TY1:3xFlag::EGFP expressed from the *wgIs638* transgene in the untreated and cisplatin-treated 1-day-old old animals. Worms were exposed for 6 h to cisplatin on plates containing 300 µg/mL cisplatin. Increased levels of ATF-7::GFP were observed in the nuclei of intestinal cells after cisplatin treatment. Scale bar: 100 µm (upper panels) and 10 µm (bottom panels). White squares in the upper panels mark the area magnified in the bottom panels. Insets in the upper panels show light microscopy pictures of the same worms. In the bottom panels white arrows indicate the ATF-7::GFP signal in intestinal nuclei, and yellow arrows indicate the background intestinal autofluorescence signal. (b) The quantification of relative GFP intensity in *wgIs638* expressing animals in untreated (n=12) and cisplatin-treated 1-day-old animals (n=8). Worms were exposed for 6 h to cisplatin on 300 µg/mL cisplatin-containing plates. Box plots indicate median (middle line), 25th, 75th percentile (box) and Min to Max (whiskers). Statistical significance was determined by the independent two-sided t-test. Bars represent mean ± SD. (c) Representative bright-field photographs of *atf-7(qd22) T24B8.5p::GFP* (*agIs219*) (n=10) and *atf-7(qd22) T24B8.5p::GFP*; *ATF-7::3xFlag* (n=9) showing restoration of intestinal T24B8.5p::GFP fluorescence in *atf-7(qd22)* animals. Scale bar: 100 µm. Source data are provided as a Source Data file.

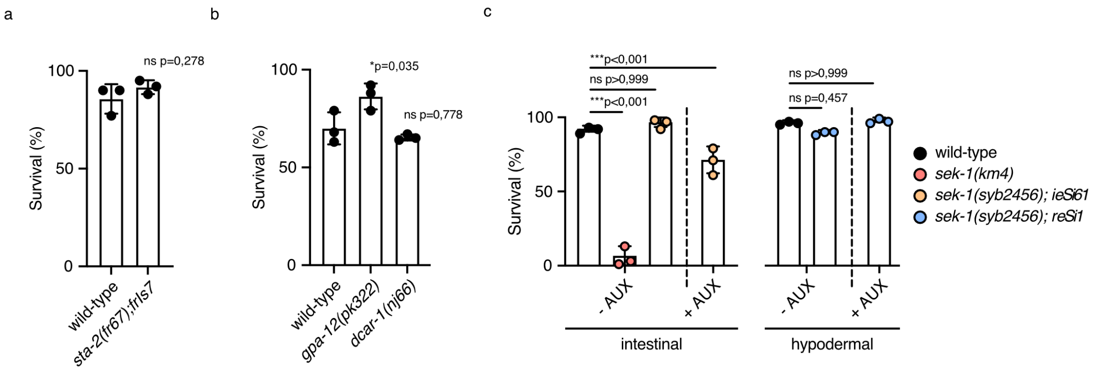

Supplementary Figure 9. Mean survival ± SD of 1-day-old adults with the indicated genotypes after 24 h cisplatin exposure at a concentration of (a) 300 µg/mL (b) 450 µg/mL Statistical significance was determined by (a) the independent two-sided t-test or (b) one-way ANOVA followed by Bonferroni post hoc correction. The experiments were performed in triplicate. (c) Mean survival ± SD of 1-day-old adults with the indicated genotypes after 24 h cisplatin (300 µg/mL) exposure without (- AUX) or with (+ AUX) 1mM auxin treatment. The *ieSi61* transgene enables auxin-dependent intestinal specific depletion of SEK-1::cMyc::AID and the *reSi1* transgene enables hypodermal specific depletion. Statistical significance was determined by the one-way ANOVA followed by Bonferroni post hoc correction (n≥50). The experiments were performed in triplicate. Source data are provided as a Source Data file.

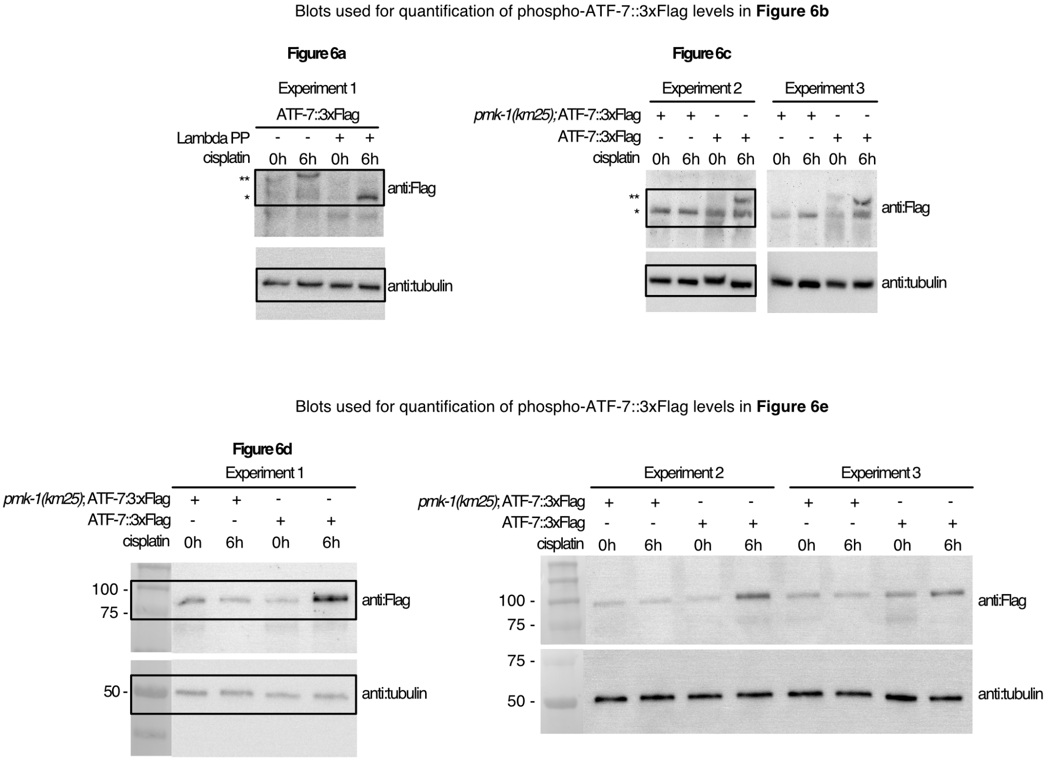

Supplementary Figure 10. Full blot images.

Blots used for quantification of IRE-1::3xFlag levels in **Figure 7e**

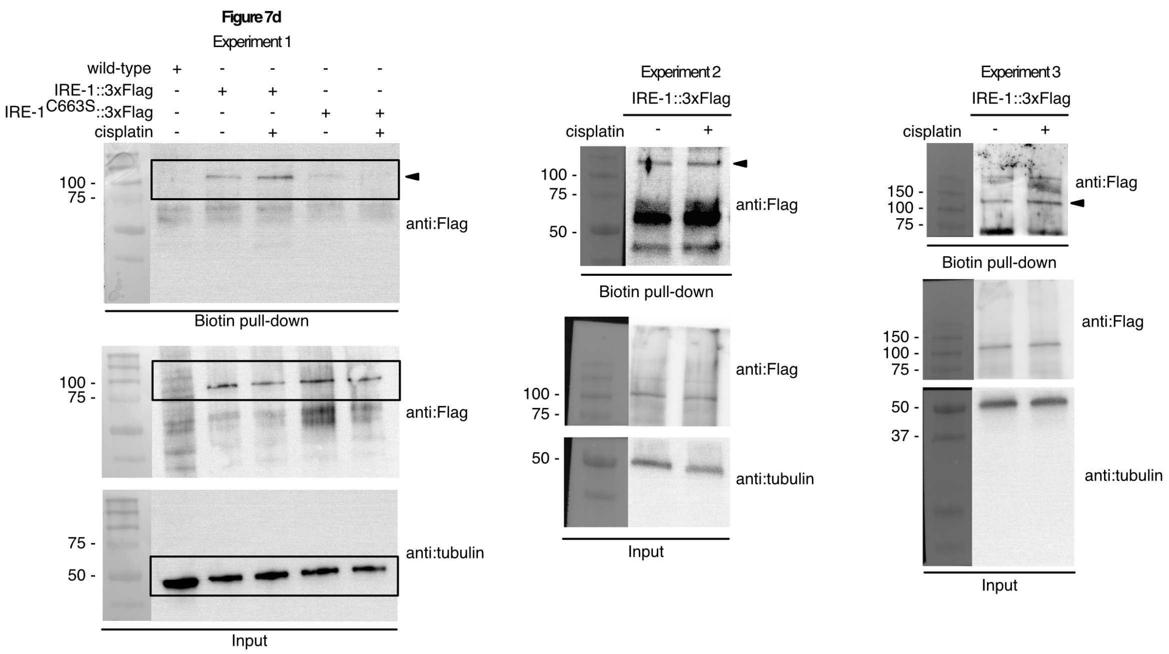

Supplementary Figure 11. Full blot images. Arrowheads indicate the band used for quantification in Figure 7e.

a

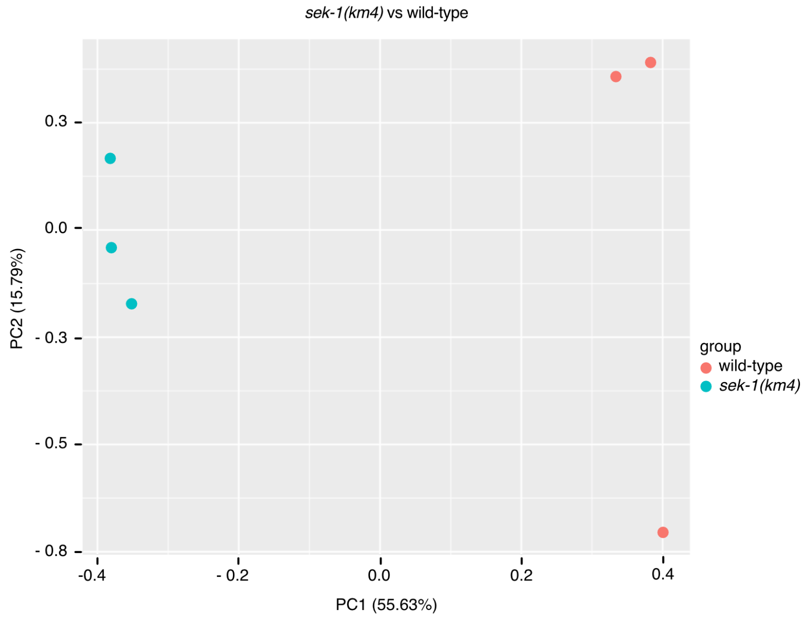

b

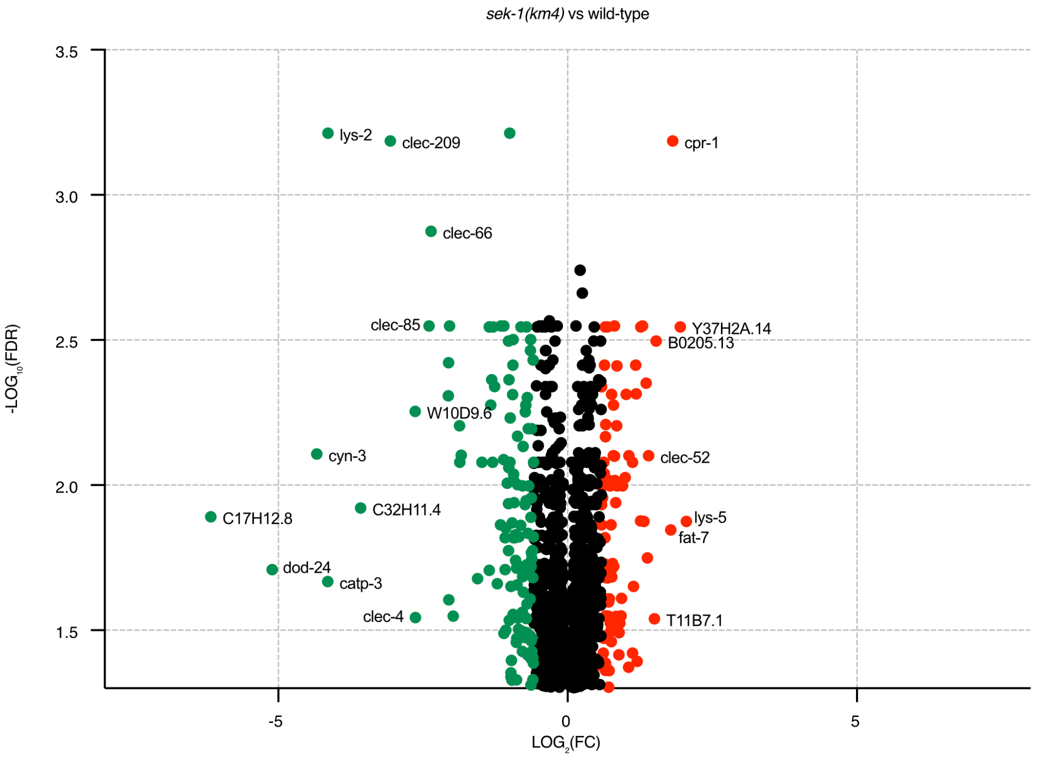

Supplementary Figure 12. (a) Principal component analysis (PCA) of the proteomics data sets from 1-day-old adult *sek-1(km4)* and wild-type animals. (b) Volcano plot of differentially abundant proteins (FDR < 0.05) in 1-day-old adult *sek-1(km4)* animals in comparison to the wild-type strain. Proteins that had a positive fold change (FC > LOG2(1.5)) after cisplatin treatment are indicated in red and negative fold change (FC < -LOG2(1.5)) after cisplatin treatment are indicated in green. FC, fold change. FDR, false discovery rate. For details see Supplementary Data 2.

a

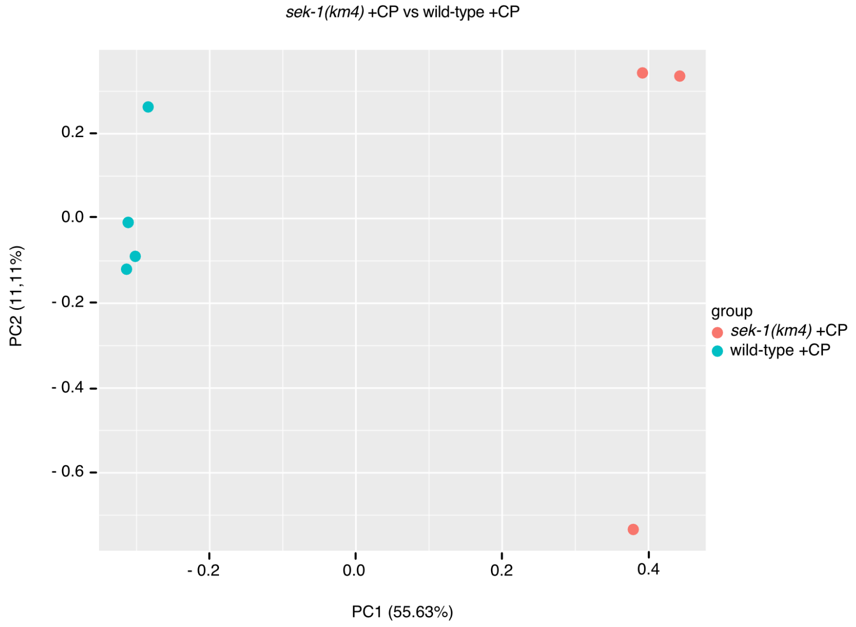

b

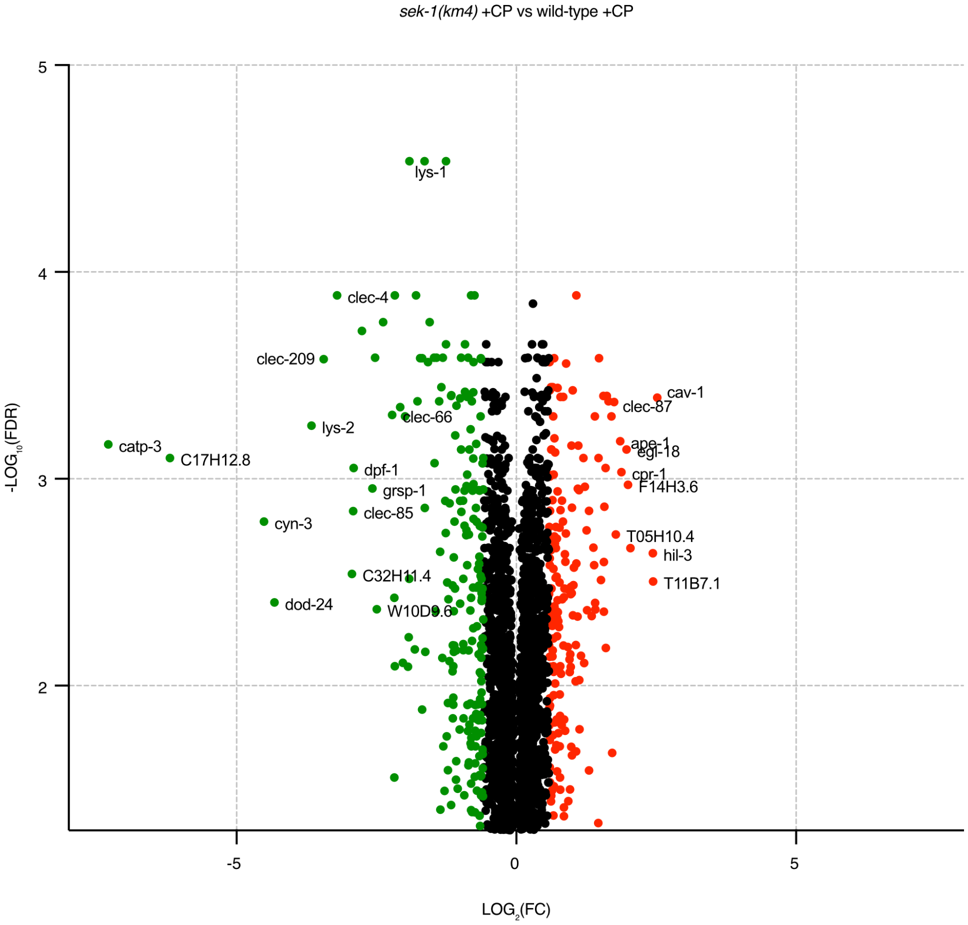

Supplementary Figure 13. (a) Principal component analysis (PCA) of the proteomics data sets from 1-day-old adult *sek-1(km4)* and wild-type animals treated with cisplatin (300 µg/mL for 6 h). (b) Volcano plot of differentially abundant proteins (FDR<0.05) in treated with cisplatin 1-day old adult *sek-1(km4)* animals in comparison to the treated with the cisplatin

wild-type strain. Proteins that had a positive fold change (FC > LOG2(1.5)) after cisplatin treatment are indicated in red and negative fold change (FC < -LOG2(1.5)) after cisplatin treatment in green. FC, fold change. FDR, false discovery rate. For details see Supplementary Data 2.

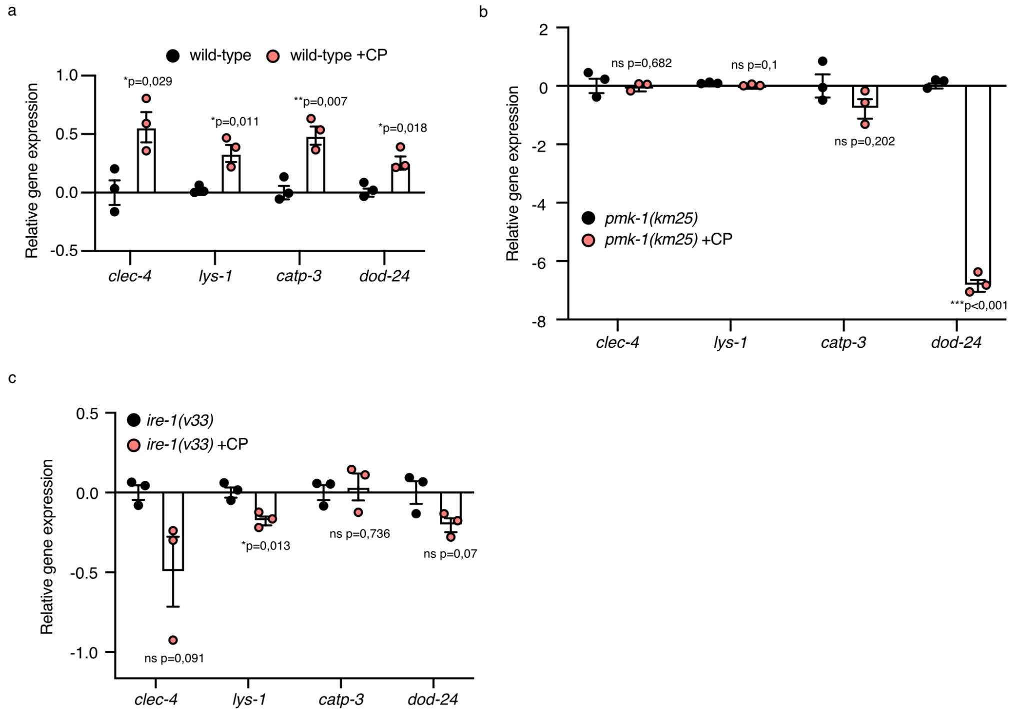

Supplementary Figure 14. (a-c) Relative gene expression in 1-day-old adult animals of the indicated genotypes without or with (+CP) cisplatin treatment. Worms were exposed for 3.5 h to 300 µg/mL cisplatin solution. Statistical significance was determined by the independent two-sided t-test. The experiment was performed in triplicate. F44B9.5 was used as a normalizing control. Bars represent mean ± SEM. Source data are provided as a Source Data file.

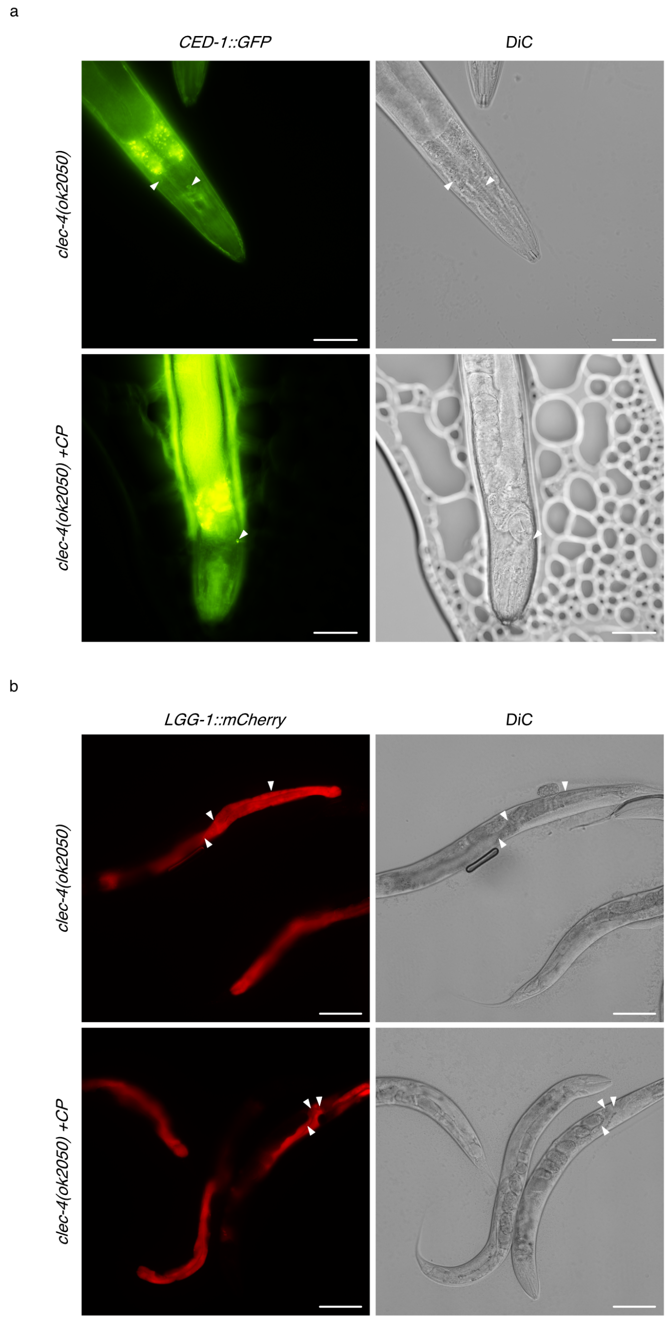

Supplementary Figure 15. Representative fluorescent pictures of (a) CED-1::GFP expressed from the *enIs7* transgene and (b) intestinal LGG-1:mCherry expressed from the *svIs143* transgene in untreated and cisplatin-treated (+CP) 1-day old *clec-4(ok2050)* animals. Worms were exposed for 18 h to cisplatin on 450 µg/mL cisplatin-containing plates. Scale bar: (a) 10 µm, (b) 100 µm. White arrows indicate apoptotic bodies in (a) and intestinal autophagosome puncta in (b).

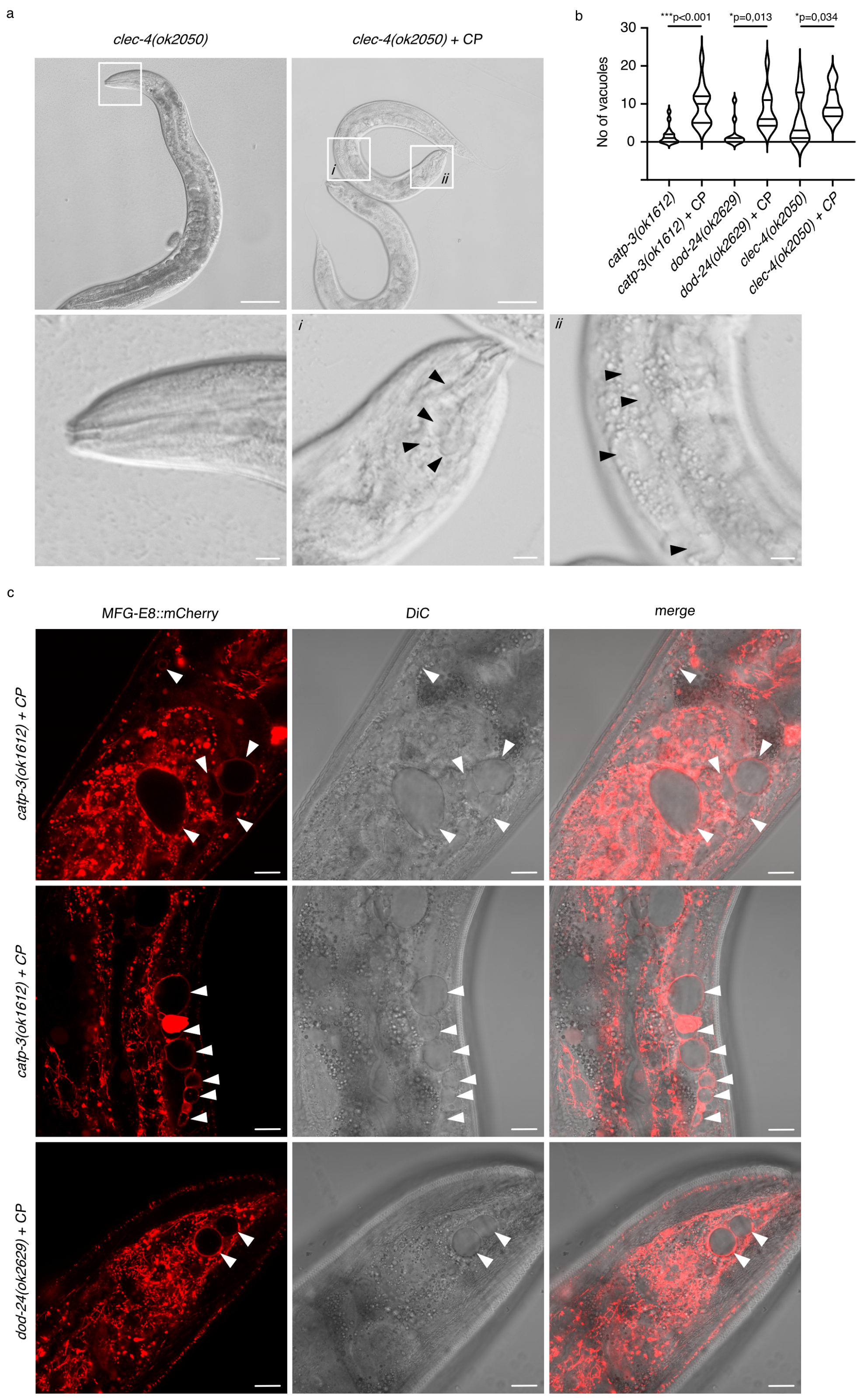

Supplementary Figure 16. (a) Representative bright-field photographs of *clec-4(ok2050)* in untreated and cisplatin-treated (+CP) 1-day-old animals. Worms were exposed for 18 h to cisplatin on 450 µg/mL cisplatin-containing plates. Increased number of vacuoles after cisplatin treatment was observed in the head region and intestines of the animals. Scale bar: 100 µm (upper panels) and 10 µm (bottom panels). White squares in the upper panels mark the area magnified in the bottom panels. In the bottom panels, black arrows indicate the

vacuoles in (*i*) head region and (*ii*) mid-body region. (b) The estimation of the number of vacuoles in the head region of untreated (*catp-3(ok1612)*: n=15; *dod-24(ok2629)*: n=10; *clec-4(ok2050)*: n=15) and cisplatin-treated (300 µg/mL for 18 h) 1-day old animals (*catp-3(ok1612)*: n=13; *dod-24(ok2629)*: n=12, *clec-4(ok2050)*: n=14). with indicated genotypes. Statistical significance was determined by the Kruskal-Wallis test followed by Dunn's post hoc correction. (c) Representative fluorescent confocal micrographs and bright field pictures of *dod-24(ok2629);mfg-e8::mCherry* (head and mid-body region) *catp-3(ok2629);mfg-e8::mCherry* (head region) in cisplatin-treated (+CP) 1-day old animal. Worms were exposed for 18 h to cisplatin on 450 µg/mL cisplatin-containing plates. White arrows mark the vacuoles whose membranes accumulate MFG-E8::mCherry expressed from the *enIs74* transgene. Scale bar: 10 µm.

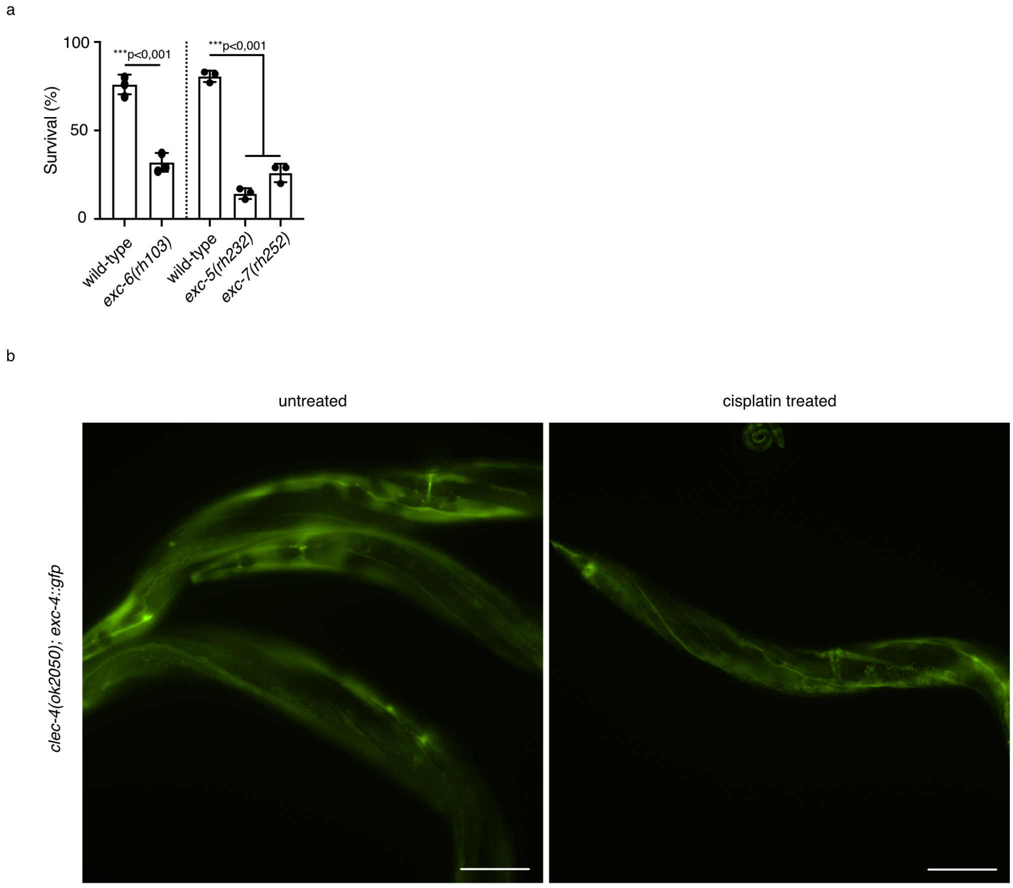

Supplementary Figure 17. (a) Mean survival ± SD of 1-day-old adults with the indicated genotypes after 24h cisplatin (450 µg/mL) exposure. Statistical significance was determined by one-way ANOVA followed by Bonferroni post hoc correction. The experiments were performed in triplicate. (b) Representative fluorescent pictures of *clec-4(ok2050);exc-4::gfp* (*otEx671*) in untreated and cisplatin-treated (+CP) 1-day old animal. Worms were exposed for 18 h to cisplatin on 450 µg/mL cisplatin-containing plates. Scale bar: 100 µm.

Supplementary Table 1. Oligonucleotides sequences used in this study.

Gene name	Forward (F) /Reverse (R)	Sequence
hsp-4	F	GGAGGATCAACCAGAATTCC
	R	GGTTGATTCCACGAGATGG
clec-4	F	AGAATTCTATTGACAACCGAGC
	R	CGAAACAGTAAAGTCCCATCC
lys-1	F	TTCGGATCTTTCAAGAAG
	R	TGGGATTCCAACAACGTA
dod-24	F	ACCGAGCCAGGAGGTTATCT
	R	TCCCGATGTTGATTTTGACC
catp-3	F	CTGTCACCGATTTGTGGTAC
	R	CCATCCAATTGTGTTGATTGC
gcs-1	F	CCAATCGATTCCTTTGGAGAC
	R	CGATGAGACCTCCGTAAGG
gst-4	F	GTCTATCACAAGATACTTGGCA
	R	TAATATGATCAGCGTCACTTCC
gst-30	F	CTCAAGTCGACTATGCCGT
	R	AGAAATTGCATGGGATTGTCC
gst-38	F	GTGGGAGAAGTTCAAAGCG
	R	TACCTCGAGCATTGGAAGC
C32H11.1	F	TAGATACTAAGGCTGCTGGA
	R	TGATAGAACATCCTCTTGTTCG
F49F1.6	F	ACGCTGTTAAGATACAATGCC
	R	GTCTGGGAGGAGCTAATGTC
F56D6.2	F	TGCTCTTTGATCTTGCCTC
	R	TCCATCCAATGAAATGCGA
K08D8.5	F	TTCCATACAATGCAAACGTG
	R	AATCCTTGGGTGTAGTTTCC
F35E12.5	F	TCTTACTCTCCTGAACTTGTACC
	R	TCGTACTTCTTCACCGCAG
F08G5.6	F	ACATTCATCAACAGTCGTTCTG
	R	CGTGGAAACTAGGAAACTTGTG
T24B8.4	F	CTATCGTCAACCCAGAACC
	R	CAAACGAGATCCGATACGG
atf-7	F	AAGGAGCAATATGTCATCTTCG
	R	CTCGTTGAAGCAGTTGATCC
