# Supplementary material for: Cisplatin toxicity is counteracted by the activation of the p38/ATF-7 signaling pathway in post-mitotic C. elegans
Source: Nat Commun. 2023 May 20;14:2886. doi: 10.1038/s41467-023-38568-5 (PMC10199892; doi:10.1038/s41467-023-38568-5)
Supplement: Supplementary file 3 — Description of Additional Supplementary Files [file 41467_2023_38568_MOESM3_ESM.pdf]

Description of Additional Supplementary Files

Supplementary Data 1. List of differentially expressed proteins. Differentially expressed proteins were identified by using a two-sample t-test on log-transformed data. To control for multiple testing, the Benjamini-Hochberg procedure was used. Proteins with an FDR value < 0.05 were considered differentially expressed. For each, the following are provided: UniProt accession, description, gene name, molecular weight (kDa), coverage (%), number of peptides, number of unique peptides, abundance ratio, mean abundance ratio, fold change (fc), p-value, false discovery rate. Worms for this experiment were exposed for 6 h to cisplatin (CP) on plates containing 350 µg/mL cisplatin. Sheet 1: wild-type +CP vs wild-type; Sheet 2: *sek-1(km4)* vs wild-type; Sheet 3: *sek-1(km4)*+CP vs wild type.

Supplementary Data 2. Reactome enrichment analysis and gene ontology enrichment and classification for wild-type samples. List of enriched pathways (FDR < 0.05) (Sheet 1) and biological processes (Sheet 2) upon cisplatin treatment in wild-type animals. For Reactome enrichment analysis, the following are provided: ID number, description of the pathway, pathway name, gene ratio, bg ratio, p-value, adjusted p-value, q-value, genes ID, and count of proteins. Biological process GO terms are listed with associated GO biological process name, # of genes within the respective term, # of input genes that were annotated to the respective term, fold enrichment, and p-values. For Reactome enrichment analysis the hypergeometric model was implement to assess whether the number of selected genes associated with Reactome pathway is larger than expected. The p values were calculated based the hypergeometric model.

Supplementary Data 3. Comparison of differentially expressed proteins across the samples with and without cisplatin treatment. The following are provided: UniProt accession, description, and fold change (fc).

Supplementary Data 4. Cisplatin sensitivity source data. For each, the following are provided: experiment number, strain name, strain genotype, cisplatin concentration (µg/mL), number of dead animals, number of alive animals, and survival (%).

Supplementary Data 5. *C. elegans* strains used in this study. Sheet 1: for each, the following are provided: strain name, strain genetic background, transgene name, notes, and references.
