# Supplementary material for: Cisplatin toxicity is counteracted by the activation of the p38/ATF-7 signaling pathway in post-mitotic C. elegans
Source: Nat Commun. 2023 May 20;14:2886. doi: 10.1038/s41467-023-38568-5 (PMC10199892; doi:10.1038/s41467-023-38568-5)
Supplement: Supplementary file 9 — Reporting Summary [file 41467_2023_38568_MOESM9_ESM.pdf]

nature portfolio

Corresponding author(s): Gautam Kao, Peter Naredi

Last updated by author(s): Mar 31, 2023

Reporting Summary

Nature Portfolio wishes to improve the reproducibility of the work that we publish. This form provides structure for consistency and transparency in reporting. For further information on Nature Portfolio policies, see our Editorial Policies and the Editorial Policy Checklist.

Statistics

For all statistical analyses, confirm that the following items are present in the figure legend, table legend, main text, or Methods section.

n/a	Confirmed	
☐	☒	The exact sample size (*n*) for each experimental group/condition, given as a discrete number and unit of measurement
☐	☒	A statement on whether measurements were taken from distinct samples or whether the same sample was measured repeatedly
☐	☒	The statistical test(s) used AND whether they are one- or two-sided<br>*Only common tests should be described solely by name; describe more complex techniques in the Methods section.*
☐	☒	A description of all covariates tested
☐	☒	A description of any assumptions or corrections, such as tests of normality and adjustment for multiple comparisons
☐	☒	A full description of the statistical parameters including central tendency (e.g. means) or other basic estimates (e.g. regression coefficient) AND variation (e.g. standard deviation) or associated estimates of uncertainty (e.g. confidence intervals)
☐	☒	For null hypothesis testing, the test statistic (e.g. *F*, *t*, *r*) with confidence intervals, effect sizes, degrees of freedom and *P* value noted<br>*Give P values as exact values whenever suitable.*
☒	☐	For Bayesian analysis, information on the choice of priors and Markov chain Monte Carlo settings
☒	☐	For hierarchical and complex designs, identification of the appropriate level for tests and full reporting of outcomes
☒	☐	Estimates of effect sizes (e.g. Cohen's *d*, Pearson's *r*), indicating how they were calculated

Our web collection on statistics for biologists contains articles on many of the points above.

Software and code

Policy information about availability of computer code

Data collection	No software was used for data collection.
Data analysis	ImageJ (version 2.0.0-rc-69/1.52p) was used to quantify pixel intensity.<br>Prism 7 software (GraphPad software, La Jolla, Version 9.5.1 (528)) was used for statistical analysis

For manuscripts utilizing custom algorithms or software that are central to the research but not yet described in published literature, software must be made available to editors and reviewers. We strongly encourage code deposition in a community repository (e.g. GitHub). See the Nature Portfolio guidelines for submitting code & software for further information.

Data

Policy information about availability of data

All manuscripts must include a data availability statement. This statement should provide the following information, where applicable:

- Accession codes, unique identifiers, or web links for publicly available datasets
- A description of any restrictions on data availability
- For clinical datasets or third party data, please ensure that the statement adheres to our policy

All data is available in the main text or supplementary materials. Source data are provided with this paper. The mass spectrometry proteomics data have been deposited to the ProteomeXchange Consortium via the PRIDE partner repository with the dataset identifier PXD033377.

Human research participants

Policy information about studies involving human research participants and Sex and Gender in Research.

Reporting on sex and gender	This information has not been collected
Population characteristics	This information has not been collected.
Recruitment	This information has not been collected.
Ethics oversight	This information has not been collected.

Note that full information on the approval of the study protocol must also be provided in the manuscript.

Field-specific reporting

Please select the one below that is the best fit for your research. If you are not sure, read the appropriate sections before making your selection.

- [x] Life sciences
- [ ] Behavioural & social sciences
- [ ] Ecological, evolutionary & environmental sciences

For a reference copy of the document with all sections, see nature.com/documents/nr-reporting-summary-flat.pdf

Life sciences study design

All studies must disclose on these points even when the disclosure is negative.

Sample size	No sample size calculation was performed. For cisplatin sensitivity assays sample sizes of at least 20 worms per repetition was chosen to provide a sufficient sample size or statistical analysis. The qPCR ans Western blot experiments were performed in triplicates as standard method. For worms fluorescent quantification at lest 10 worms per conditions were chosen in order to provide a sufficient sample size for statistical analysis.
Data exclusions	No data were excluded from the analyses.
Replication	All experiments were performed in at least triplicates to confirm the reproducibility of the experimental findings and all attempts were successful.
Randomization	Randomization was not relevant for the study. Use of the C. elegans populations do not require the randomization.
Blinding	Blinding was not relevant for the study. Use of the C. elegans populations do not require the blinding

Reporting for specific materials, systems and methods

We require information from authors about some types of materials, experimental systems and methods used in many studies. Here, indicate whether each material, system or method listed is relevant to your study. If you are not sure if a list item applies to your research, read the appropriate section before selecting a response.

Materials & experimental systems

n/a	Involved in the study	
[ ]	[x]	Antibodies
[x]	[ ]	Eukaryotic cell lines
[x]	[ ]	Palaeontology and archaeology
[ ]	[x]	Animals and other organisms
[x]	[ ]	Clinical data
[x]	[ ]	Dual use research of concern

Methods

n/a	Involved in the study	
[x]	[ ]	ChIP-seq
[x]	[ ]	Flow cytometry
[x]	[ ]	MRI-based neuroimaging

Antibodies

Antibodies used	1) anti-phospho-p38 MAPK (Thr180/Thr182) Rabbit mAb (D3F9, Cell Signalling), Cat# 9215S, LOT:7<br>2) anti-tubulin (T5168, Sigma), Cat# T5168, LOT:00000089494<br>3) anti-Flag (M2, Sigma), Cat# F1804, LOT:SLCJF4933<br>4) anti:total PMK-1 MAPK Rabbit pAb 1:1000 from the Pukkila-Worley lab

Validation	All antibiodies were validated by the manufacturer and validtation statements are available at the manufacturer's website:<br>1) anti-phospho-p38 MAPK (Thr180/Thr182) Rabbit mAb (D3F9, Cell Signalling), https://www.cellsignal.com/products/primary-antibodies/phospho-p38-mapk-thr180-tyr182-3d7-rabbit-mab/9215?site-search-type=Products&N=4294956287&Ntt=phospho-p38+mapk+%28thr180%2Ftyr182%29+&fromPage=plp<br>2) anti-tubulin (T5168, Sigma): https://www.sigmaaldrich.com/SE/en/product/sigma/t5168?context=product; 2) Relevant citation: Solinger JA et al. (2010) PLoS Genet. 6(1):e1000820<br>3) anti-Flag (M2, Sigma): https://www.sigmaaldrich.com/SE/en/product/sigma/f1804?context=product<br><br>Anti:total PMK-1 MAPK Rabbit was validated by the Pukkila-Worley lab where it was manufactured. Validation is available here: https://doi.org/10.1371/journal.pgen.1007935.

Animals and other research organisms

Policy information about studies involving animals; ARRIVE guidelines recommended for reporting animal research, and Sex and Gender in Research

Laboratory animals	Caenorhabditis elegans hermaphrodite were used in the study in the 1-day old adult stage. Strains used in the study are listed in Supplemenatry Data 5. All C. elegans strains used in the study are available from the authors or from the standard commercial sources: Caenorhabditis Genetic Center (CGC) (www.cgc.umn.edu).
Wild animals	This study did not involve wild-type animals
Reporting on sex	This information has not been collected.
Field-collected samples	This study did not involve samples collected from the field.
Ethics oversight	No ethical approval was required.

Note that full information on the approval of the study protocol must also be provided in the manuscript.
